# Utilisation of dental services by refugees in Germany: Results of the population-based RESPOND survey

**DOI:** 10.25646/11844

**Published:** 2024-01-17

**Authors:** Kayvan Bozorgmehr, Maren Hintermeier, Louise Biddle, Claudia Hövener, Nora Gottlieb

**Affiliations:** 1 AG 2 Population Medicine and Health Services Research, School of Public Health, Bielefeld University, Germany; 2 Section for Health Equity Studies and Migration, Department of General Practice and Health Services Research, Heidelberg University Hospital, Germany; 3 German Institute for Economic Research (DIW Berlin); 4 Robert Koch Institute, Berlin, Germany, Department of Epidemiology and Health Monitoring

**Keywords:** HEALTH MONITORING, REFUGEES, SURVEY, UTILISATION, DENTAL CARE

## Abstract

**Background:**

The utilisation of outpatient dental services is an important indicator for monitoring healthcare provision in Germany. In the general population, the 12-month prevalence of dental service utilization is 82.2 %. For refugees, this indicator has hardly been measured, although studies suggest an objectively high need for dental care.

**Methodology:**

As part of the population-based cross-sectional RESPOND study (2018), self-reported health and healthcare, including the use of dental services, was assessed in three representative, random samples of refugees residing in reception and shared accommodation centres in Baden-Württemberg and Berlin.

**Results:**

The indicator was available for 68.8 % (594) of the 863 surveyed refugees. Overall, 38.2 % of the respondents stated that they had utilised dental services in the previous 12 months, whereas 41.4 % had never used any dental care in Germany.

**Conclusions:**

The utilisation of dental services among refugees is very low compared to the level of utilisation in the general population. It reflects a discrepancy between access and needs.

## Introduction

The utilisation of outpatient dental services is an important indicator for monitoring healthcare provision. It is routinely measured nationwide in the general population as part of the ‘German Health Update (GEDA)’ study. The results of the most recent survey wave of the study (GEDA 2019/2020-EHIS) showed that an average of 82.2 % of the population had a dental examination in the 12 months prior to the survey. The highest prevalence of utilisation was found in the population aged 45 to 64 years (84.4 %), in Saxony (87.4 %), and among people with a high educational level (87.0 %). The lowest prevalence was recorded in the age group 80 years and older (71.5 %), in Hesse (78.1 %), and among people with a low level of education (75.0 %) [1]. However, the indicator is not measured for all population groups. For example, refugees (Infobox 1) have so far been insufficiently included in health surveys [2]. Public debates on the utilisation of medical services by refugees and potential underuse, overuse or misuse should be based on evidence. International studies [3, 4] and studies from Germany [5–7] consistently indicate that the oral health status of refugees is poor. Although the number of carious, missing or filled permanent teeth among refugees aged 18 to 34 years is moderate, there are high levels of unmet needs and about a quarter of the study respondents present with complications [6]. Oral health declines in older age groups, with highest unmet needs for dental care reported in the group of 35- to 44-year-old refugees [6].

**Infobox 1 Refugees**

This article refers to ‘refugees’ as all persons who have filed an application for asylum in Germany with the Federal Office for Migration and Refugees (BAMF) – regardless of the outcome of the asylum application. Individuals who have been resettled in Germany as quota refugees by the UN Refugee Agency (UNHCR) in accordance with the Geneva Refugee Convention or who have been granted temporary protection status on the basis of European Union legal norms are also considered refugees.

In Germany, §§ 4 and 6 of the Asylum Seekers’ Benefits Act (AsylbLG) regulate refugees’ entitlement and access to medical services, including dental care. Dental prosthetic treatments, in particular, are regulated by § 4 (1) (Infobox 2), which stipulates that benefits be granted on a case-by-case basis and only if treatment cannot be postponed for medical reasons [8]. Benefits beyond this level can be granted (in accordance with § 6 AsylbLG) on a discretionary basis and upon request to the competent authority. The above laws apply to asylum-seekers whose asylum proceeding is pending, whose asylum application was declined, and who were granted a ‘toleration’ (non-deportability) during their first 18 months of residence in Germany [9]. The government plans to extend this period to 36 months in the future [10]. Refugees with Ukrainian citizenship are exempt from the above regulations and entitled to the standard benefits of statutory health insurance in accordance with the Social Code Book (SGB) II and XII [11].

From a healthcare-epidemiological perspective, the restrictive legal regulations of the AsylbLG and the numerous other barriers hampering refugees’ access to health care [12] raise questions concerning the actual utilisation of dental services by this population group. This paper is the first to report population-based data on the utilisation of dental services by refugees in Germany, while considering age, sex, legal status and length of stay in Germany.

## Indicator

The data presented here were collected as part of the population-based health surveys among refugees of the project ‘Improving Regional Health System Responses to the Challenges of Forced Migration’ (RESPOND, www.respond-study.org), which was funded by the Federal Ministry of Education and Research (BMBF). The database comprises three samples of a total of 863 refugees [13], which were drawn from reception and shared accommodations centres in Baden-Württemberg [14] and Berlin [15] in 2018 in a cross-sectional, randomised study design. Sampling, recruitment and survey instruments were virtually identical in both federal states [14, 15]. In both settings, a total of approximately 3 % of all 2,017 shared accommodation centres (corresponding to 81 accommodation centres) were randomly selected and, if possible, all adult residents were personally invited by multilingual field teams to take part in the survey. The six state-level reception centres (LEA) were not selected at random, but according to geographical criteria (widespread spatial distribution within the respective federal state) and occupancy rates (facilities with high occupancy rates in order to generate sufficient case numbers). However, the actual respondents in the LEAs were selected randomly by drawing a random sample from all occupied rooms, which was then used to select the study participants [14]. The response rate of the survey was 30.5 % [13]. The written survey was conducted using a questionnaire in nine languages (Albanian, Arabic, German, English, Persian, French, Russian, Serbian and Turkish). The questionnaire was based on standardised and established instruments as well as a comprehensive development and adaptation process (for details of the methodology, see [14]). The majority (96 %) of respondents were surveyed between January 2018 and November 2018 [13].

**Infobox 2 Excerpt from the Asylum Seeker’s Benefits Act (AsylbLG)**

§ 4 Benefits related to illness, pregnancy and birth

(1) The medical and dental treatment required for treatment of acute illnesses and painful conditions, including the supply of medicines, dressings and other services required for recovery, improvement or alleviation of illnesses or consequences of illness, shall be provided. Vaccinations in accordance with §§ 47, 52 paragraph 1 sentence 1 of the Twelfth Book of the German Social Code for purposes of prevention and early detection of illnesses and medically required preventive medical check-ups shall be provided. Dental prostheses shall be provided only in as far as this cannot be postponed in the individual case for medical reasons.

§ 6 Other benefits

(1) Other benefits may be granted in particular cases, if they are essential in the individual case to ensure subsistence or health, if they are required to meet the special needs of children or to fulfil an administrative obligation to cooperate. The benefits are to be granted as benefits in kind or, in special circumstances, as cash benefits.

Source: Federal Office of Justice

https://www.gesetze-im-internet.de/asylblg

The utilisation of (dental) medical care was assessed by means of instruments from the European Health Interview Survey (EHIS). The exact phrasing was ‘When was the last time you consulted the following medical practitioners in Germany on your own behalf?’, followed by several specialist medical disciplines, including ‘dentist’ as one of the response options. The wording of the question differed slightly from the wording of the original question used in the EHIS and GEDA (‘When was the last time you saw a dentist, orthodontist or other dental specialist for counselling, examination or treatment for yourself?’). Accordingly, GEDA 2019/2020-EHIS does not provide a list of doctors to choose from, but instead asks individual questions for each group of doctors. Moreover, the question in GEDA 2019/2020-EHIS refers to a visit to ‘a dentist, orthodontist or other dental specialist’, which must be considered in the interpretation of the results of the different surveys.

In RESPOND, health care utilisation was recorded using four response options (‘less than 12 months ago’, ‘12 months ago or longer, ‘never’ and ‘I don’t know’ (‘I’ is missing)). Information in the ‘I don’t know’ category was treated as missing values in the present analysis, as it does not provide any information about the actual utilisation behaviour. In addition to basic socio-demographic characteristics such as sex (male, female, diverse) and month and year of birth for calculation of the age, the legal status was recorded with the following question: ‘What is your current legal status in Germany?’ Four response options (‘options’ missing) allowed for a simplified description of the respondents’ legal status in the survey results, by distinguishing between pending and completed asylum procedures and their different outcomes (‘ongoing asylum process – quotation marks missing, ‘completed asylum process – asylum granted’, ‘completed asylum process – toleration (‘Duldung’)’, ‘completed asylum process – rejected and asked to leave the country’). The length of stay was calculated from the reported date of entry (month/year) into Germany (‘When did you arrive in Germany?’) and was sub-divided into two categories (‘0 to 12 months’, ‘13 to 36 months’).

This paper reports the 12-month prevalence (in %) of the ‘utilisation of dental care’ indicator overall as well as by sex, age, legal status and length of stay in Germany. Age was recorded in years and months, but categorised into two groups (18 – 30 years, > 30 years of age) due to low case numbers. The data set was weighted based on information on sex, age and region of origin for the two federal states in order to improve the estimates for the study population [13]. Missing values were not imputed, but only valid values were included in the analysis.

## Results and interpretation

Valid data for the utilisation of dental care were available for 594 of the total of 863 individuals surveyed (68.8 % of the sample). Out of this total population (N = 594), 29.0 % were women and 42.1 % were aged between 18 and 30 years. Regarding the legal status, 43.4 % reported an ongoing asylum procedure, while 26.3 % had been granted asylum. The asylum application of 17.5 % of the respondents had been rejected, meaning that they were either tolerated or asked to leave the country. A total of 25.4 % of the study population had been staying in Germany for 0 to 12 months.

The proportion of those who had visited a dentist in the last 12 months for treatment, counselling or examination was 38.2 %. At the same time, 41.4 % of surveyed persons stated that they had never visited a dentist in Germany (Table 1).

The prevalence of utilisation did not vary by sex. The 12-month prevalence of utilisation was highest in the age group over 30 years (44.4 %), while the proportion of those who had never visited a dentist was highest among the 18 to 30-year-olds at almost 50 %. As regards associations with legal status, the 12-month prevalence of utilisation was highest among respondents who had either been granted asylum and among respondents who were tolerated or asked to leave the country at the time of the survey, at over 40 % each. At 46.4 %, the proportion of those who had never visited a dentist in Germany was highest among those whose asylum procedure was ongoing. However, these differences appear to be random (given broad and overlapping 95 % CI). A shorter length of stay was associated with a lower prevalence of utilisation: In the group of respondents, who had been residing in Germany for 0 to 12 months, more than two thirds (71.8 %) had not visited a dentist in Germany – compared to one third (33.1 %) of respondents who had been living in Germany for longer.

These analyses do not allow for conclusions about group-related differences, as these may be influenced by other characteristics such as a different age composition or length of stay in Germany. A more in-depth analysis of any differences in the prevalence of dental care utilization would have required larger samples.

Nevertheless, the prevalence data from this random sample provide a valid estimate of the utilisation of dental care among refugees in two federal states. A direct comparison between the utilisation values collected here (Table 1) and the values collected for the general population by the GEDA 2019/2020-EHIS survey, however, is possible only to a limited extent and allow an approximation at best. One reason is the different wording of the questionnaire items. The question is less specific in the general population and also includes orthodontists and other specialists. Moreover, differences in sampling and in the geographical location of the surveyed populations must be considered. For example, the sample of refugees was drawn in only two federal states. Given the considerable heterogeneity in the organisation of health care for refugees [13, 16], the estimates of the utilisation of dental care may not be applicable to all federal states.

Daring to make such comparison from the perspective of the ‘best available data’, the values for dental care utilisation among refugees are clearly lower than those among the general population, as per the GEDA study. Accordingly, in Berlin and Baden-Württemberg, 82.1 % (95 % CI: 79.2 – 84.8) and 83.2 % (95 % CI: 80.9 – 85.3) of the population, respectively, reported to have sought dental care in the previous 12 months [1]. Hence, a difference of more than 35 percentage points lies between this value and the highest level of utilisation in the population of refugees surveyed with similar methods in the two federal states. The lowest prevalence of utilisation of dental care in the age group over 80 years in the German population is 71.5 % (95 % CI: 67.9 – 74.8) and thus still more than 25 percentage points higher than the utilisation by the surveyed refugees. In addition, a large proportion (> 40 %) of the surveyed refugees had never been to a dentist in Germany.

Inequalities in dental care utilization of this magnitude, and the associated structural health care inequalities resulting from the AsylbLG, should be discussed as contradictions to the constitutional principles of non-discrimination, equality and human dignity, even if there may be differences in the age structure and dental needs between refugees and the general population that cannot be examined in more detail on the basis of the available data. This is because the medical need for dental care arises primarily from the recommendation of a biannual preventive visit for adults [17]. Given that available studies on the oral health of refugees indicate a high need for dental care – in some cases higher compared to the general population [6] – the utilization pattern presented here does not appear to reflect needs-based health care provision. From the perspective of health promotion and prevention, a lack of prophylactic dental care and a restriction of care to painful and urgent conditions, combined with a high need [6], is detrimental. Untreated oral health issues can not only significantly impact quality of life, but also lead to secondary complications in other organ systems [18]. In addition to the health consequences experienced by the afflicted individuals, a lack of prevention can therefore be associated with higher follow-up costs for the health care system as well [19, 20]. Accordingly, one of the few studies on the dental health of refugees in Germany found that the costs of conservative treatment of carious teeth among refugees is almost twice as high when pain is already present as compared to treatment before pain manifests [5]. This calls into question whether the AsylbLG with its restrictive criteria for granting health benefits (such as the ‘non-postponability’ of treatment for medical reasons), actually fulfils its purported policy goal of reducing healthcare expenditures.

Answering such critical healthcare-related questions will require a sustainable improvement of the data on migration and health [2]. The health needs of and provision of health care for refugees should be better reflected in existing health information systems [21]. This can not only help to uncover social and structural inequalities [22] in healthcare, but also help objectively counter fact-free discussions on migration and health.

## Key statement

- No valid data are available on the utilisation of dental services among refugees.
- The population-based RESPOND survey was conducted in two federal states in Germany to determine refugees’ use of health care, including dental services.
- A high proportion (41.4 %) of refugees in Germany had never been to the dentist. Utilisation was significantly lower than in the general population, regardless of age, sex, and legal status.
- The 12-month prevalence of a dental visit for the purposes of a consultation, examination or treatment was 38.2 % among refugees in 2018.

## Figures and Tables

**Table 1:** Prevalence of the utilisation of dental services among N = 594 refugees in Berlin and Baden-Wuerttemberg (n = 172 women, n = 370 men) by sex, age, legal status and duration of stay, weighted Source: RESPOND Survey

		Less than 12 months ago		12 months ago or longer		Never	
	Number of cases (n)*	%	(95 % CI)	%	(95 % CI)	%	(95 % CI)
**Total**	**594**	**38.2**	**(32.1 – 44.6)**	**20.4**	**(16.3 – 25.2)**	**41.4**	**(34.8 – 48.4)**
**Sex**	**542**						
Women (total)	172	38.7	(30.2 – 47.9)	19.0	(13.2 – 26.6)	42.3	(34.9 – 50.1)
Men (total)	370	38.4	(31.1 – 46.3)	20.4	(15.9 – 25.7)	41.2	(32.5 – 50.4)
**Age group**	**594**						
18 – 30 years	250	32.4	(24.9 – 40.8)	18.7	(12.3 – 27.2)	48.9	(41.2 – 56.8)
> 30 years	344	44.4	(37.6 – 51.5)	22.2	(17.6 – 27.6)	33.4	(26.2 – 41.4)
**Legal status**	**518**						
ongoing asylum process	258	37.2	(28.5 – 46.9)	16.4	(11.0 – 23.7)	46.4	(39.3 – 53.6)
asylum granted	156	41.3	(29.1 – 54.6)	24.7	(17.7 – 33.3)	34.0	(22.9 – 47.3)
Tolerated or rejected and asked to leave the country	104	40.4	(27.8 – 54.3)	20.9	(12.7 – 32.5)	38.6	(24.8 – 54.7)
**Length of stay**	**481**						
0 – 12 months	151	28.2	(17.0 – 42.9)	n.a.		71.8	(57.1 – 82.9)
13 – 36 months	330	42.7	(36.1 – 49.6)	24.2	(19.2 – 29.9)	33.1	(26.1 – 40.9)

CI = confidence interval, n.a. = no answers possible. Implausible responses (n = 8) set to missing

^*^Deviations from N = 594 may arise due to missing values for sex, age, legal status and length of stay
